# Genome Regulation and Gene Interaction Networks Inferred From Muscle Transcriptome Underlying Feed Efficiency in Pigs

**DOI:** 10.3389/fgene.2020.00650

**Published:** 2020-06-23

**Authors:** Victor A. O. Carmelo, Haja N. Kadarmideen

**Affiliations:** Quantitative Genomics, Bioinformatics and Computational Biology Group, Department of Applied Mathematics and Computer Science, Technical University of Denmark, Kongens Lyngby, Denmark

**Keywords:** muscle transcriptome, pigs, feed efficiency, gene networks, candidate biomarkers

## Abstract

Improvement of feed efficiency (FE) is key for Sustainability and cost reduction in pig production. Our aim was to characterize the muscle transcriptomic profiles in Danbred Duroc (Duroc; *n* = 13) and Danbred Landrace (Landrace; *n* = 28), in relation to FE for identifying potential biomarkers. RNA-seq data on the 41 pigs was analyzed employing differential gene expression methods, gene-gene interaction and network analysis, including pathway and functional analysis. We also compared the results with genome regulation in human exercise data, hypothesizing that increased FE mimics processes triggered in exercised muscle. In the differential expression analysis, 13 genes were differentially expressed, including: *MRPS11, MTRF1*, TRIM63, MGAT4A, KLH30. Based on a novel gene selection method, the divergent count, we performed pathway enrichment analysis. We found five significantly enriched pathways related to feed conversion ratio (FCR). These pathways were mainly related to mitochondria, and summarized in the mitochondrial translation elongation (MTR) pathway. In the gene interaction analysis, the most interesting genes included the mitochondrial genes: PPIF, MRPL35, NDUFS4 and the fat metabolism and obesity genes: *AACS, SMPDL3B, CTNNBL1, NDUFS4*, and *LIMD2*. In the network analysis, we identified two modules significantly correlated with FCR. Pathway enrichment of module genes identified MTR, electron transport chain and DNA repair as enriched pathways. The network analysis revealed the mitochondrial gene group *NDUF* as key network hub genes, showing their potential as biomarkers. Results show that genes related to human exercise were enriched in identified FCR related genes. We conclude that mitochondrial activity is a key driver for FCR in muscle tissue, and mitochondrial genes could be potential biomarkers for FCR in pigs.

## Introduction

In commercial pig production, the cost of feed is the highest individual economic factor (Jing et al., 2015; Gilbert et al., 2017). Furthermore, reduction in feed consumption per unit growth is beneficial for the environment, which is a key factor in being able to maintain sustainable and resource efficient production. In this context, there have been continuous efforts to increase feed utilization efficiency in pigs through selective breeding. For the majority of Danish production pigs, breeding boars are selected at a core central facility where potential breeding boars are tested for FCR through accurate individual measurements of feed intake and growth. Most Danish production pigs are crossbred, with the maternal line being Landrace x Danbred Yorkshire, and the paternal line being Duroc. The Durocs are well known for being heavily selected for growth and efficiency, while the two other breeds have been heavily selected for litter size and piglet survival related traits.

Feed efficiency can be defined in several ways, with the main ones being Residual Feed Intake (RFI) (Koch et al., 1963) and FCR. FCR is the ratio between feed consumed and weight gain, while RFI is calculated by fitting a model with predicting feed intake from weight gain, and finding the residual of the model prediction for each animal. In general, it is reported that selection for low FCR will result in co- selection for related traits, namely growth rate and body composition (Nkrumah et al., 2007; Gilbert et al., 2017; Yi et al., 2018). In contrast, selection for RFI is more directly focused on metabolic efficiency irrespective of daily gain and growth (Nkrumah et al., 2007; Gilbert et al., 2017; Yi et al., 2018). In general, RFI and FCR are strongly correlated, with a correlation above 0.7 and both show low to medium heritability (Do et al., 2013). In general, FCR is simpler to calculate, as RFI calculation is dependent on individual population and production factors (Hoque et al., 2009; Do et al., 2013). However, in pig production, the co-selection of growth rate and body composition when selecting for FCR selection and the simplicity of calculation are desired traits. This may explain why FCR has been the main efficiency phenotype used for selection (Gilbert et al., 2017) in the pig population in Denmark and in general pig production. One can also hypothesize that FCR is more easily translatable between breeds/populations, as it is a simple dimensionless ratio, which has a simple and generally comparable interpretation. In contrast, it is more difficult simply compare RFI values across different populations or breeds. The biological and/or genetic background of FCR in pigs remains somewhat elusive (Ding et al., 2018), thus inviting for further analysis on the topic.

The key tissue in pig production is muscle, as pig carcasses are valued according to lean meat content. Skeletal muscle is a key organ in carbohydrate and lipid metabolism and plays a large part in the storage of energy from feed (Pedersen, 2013; Turner et al., 2014; Morales et al., 2017), especially as lean growth has been one of the main goals of pig breeding programs. Increased efficiency has also been positively associated with various meat quality parameters (Czernichow et al., 2010; Lefaucheur et al., 2011; Smith et al., 2011; Faure et al., 2013; Horodyska et al., 2018a), showing that improved FE can have multiple positive outcomes. There are only a few studies analyzing muscle tissue transcriptome of pigs in a FE context (Jing et al., 2015; Vincent et al., 2015; Gondret et al., 2017; Horodyska et al., 2018b), and thus our knowledge of the muscle transcriptomic background of FE is somewhat limited. In general, previous studies have relied on small samples sizes, weak statistical thresholds and categorical division of lines divergently selected for FE. This means that more studies are still needed to uncover the connection between the transcriptome and FE in muscle tissue.

It is well known that animal models have been used extensively for the study of human diseases and human physiology. There are many studies, which cannot be ethically performed on humans (e.g., gene expression studies in multiple organs), but we are able to take advantage of the similarities between animal models and human in the molecular mechanisms to gain knowledge across species. Conversely, one could use human results to gain knowledge of animal physiology.

The Kolmogorov-Smirnov test (KS test) (Massey, 1951), is a non-parametric test which can be used to test if an empirical data distribution could be generated from a reference continuous probability distribution. The test statistics is based on calculating a discrete maximum divergence between the theoretical cumulative probability distribution and the empirical cumulative probability distribution. In statistical testing, it is very common to use arbitrary significance thresholds, typically 0.05. At the same time, typical multiple testing correction methods such as Bonferroni or Benjamini-Hochberg (Benjamini and Hochberg, 1995) have no implicit way of picking thresholds, and can be overly conservative (Narum, 2006). In situations where one is more interested in group properties than individual tests, it could be advantageous to use a metric like the overall divergence calculated in the KS-test, which does not have any implicit thresholds, for selecting genes for further analysis.

In this study, we aim to characterize the transcriptomic profiles and link them to FE traits measured in Duroc and Landrace, purebred pigs, by fitting FE as a continuous trait over a full spectrum of efficiency, from high to low. The pigs in this study were all young performance tested boars, with the potential of becoming active breeding sires. Thus, none of the pigs were negatively selected for FE as in other studies (Vincent et al., 2015; Gondret et al., 2017; Horodyska et al., 2018a), making the differences in FE and their underlying causes more representative of real practical applications. We analyzed the muscle transcriptome based on several layers of statistical-bioinformatics analyses: differential expression (DE), gene-to-gene expression interaction and weighted network analysis. Pathway and functional analysis was performed based on differentially expressed genes and genes from network analysis. The rationale behind the approach was to reveal potential biomarkers that are functionally important and are predictive of FE in pigs. Dealing with complex yet subtle phenotypes can be challenging, as the signal to noise ratio can be high, and it may be impractical or costly to collect large sample sizes. Therefore, we also suggest a novel method for selecting features based on the KS-test statistic, the divergent count.

To gain more insight on the molecular and functional background of FE, we also hypothesized, that the mechanism between differences in the muscle transcriptome of breeds with different efficiency could be similar to the differences between a rested and an exercised muscle. We adapted a translational genomics approach to investigate this by comparing human data with our pig data.

## Materials and Methods

### Sampling and Sequencing

In total, 41 purebred male uncastrated pigs where sampled for this study from two breeds, with 13 Danbred Duroc and 28 Danbred Landrace pigs. All pigs were raised at a commercial breeding station at Bøgildgard owned by the pig research center of the Danish Agriculture and Food Council (SEGES). The pigs where raised from ∼7 kg until 100 kg at the breeding station. Feed intake registrations for each pig were initiated based on a minimum weight cutoff of 28 kg, and continued for a period of 40–70 days based on a combination of the viability of each pig, and a weight limit of 100 kg. Feed intake was measured via single feeder setup, which could only be accessed by one pig at a time. While corrections for feed waste are made if necessary, no correction were made in any of the data on the pigs in this study. The pig diet consisted of a feed mixture with the main ingredients being: 39% barley, 27%, wheat, 14% soybean meal, and 6% oats. All pigs were weighed at testing start and end for calculation of FCR. FCR was calculated by dividing the growth in the testing period by the feed consumption. Residual Feed Intake (RFI) was also estimated based on the residuals of the following model (Do et al., 2013):

$$DFI_{ij}=\mu +DWG_{i}+\beta_{j}$$

Where DFI is daily feed intake and DWG is daily weight gain in the period, and β is the batch effect. RFI was calculated separately for each breed, and based on data from a larger population (Duroc *n* = 59 and Landrace *n* = 50).

Muscle tissue samples from the psoas major muscle were extracted immediately post-slaughter and preserved in RNAlater (Ambion, Austin, TX, United States). Sample were kept at −25C°, as per protocol, until sequencing.

#### Sequencing

RNA extraction, library preparation and sequencing was done by BGI.^1^ The sequencing was unstranded mRNA sequencing on BGI’s own BGISEQ-500 platform, using an in-house designed protocol, with 100 base pair unstranded paired end reads. The data was sequenced using the same protocol as the 75PE protocol in Zhu et al. (2018), but with 100bp.

### Quality Control, Mapping, and Read Quantification

Reads were trimmed and adapters removed using Trimmomatic (Bolger et al., 2014) version 0.39 with default setting for paired end reads. The QC on the data was done both pre- and post-trimming using FastQC v0.11.9, with over 99% of the reads being kept on average (Supplementary Data 1). The reads were mapped using STAR aligner (Dobin et al., 2013) version 2.7.1a using default parameters with a genome index based on *Sus scrofa* version 11.1 and using ensembl annotation *Sus scrofa* 11.1 version 96 for splice site reference. Default parameters were used for mapping except for the addition of read quantification during mapping using the –quantMode GeneCounts setting. All statistic for the reads can be found in Supplementary Data 1.

### Differential Expression Analysis

To analyze the relationship between FCR and gene expression, we applied the following overall model, and implemented it using several different methods:

$$y_{ijklm}=\mu +\beta_{1_{i}}\left(FCR\right)+\beta_{2_{j}}\left(RIN\right)+\beta_{2_{k}}\left(age\right)+BR_{l}$$

(1) $$+BA_{m}+\epsilon$$

y = normalized read counts

β_1_ = *regression coefficient of feed conversion rate*

β_2_ = *regression coefficient of RIN (RNA Integrity value)*

β_3_ = *regression coefficient of Slaugter Age (days)*

BR = effect size of Breed

BA=effect size of Batch

RNA integrity value (RIN) should be corrected for, as it affects expression, and the most appropriate way to correct this is to include it in the model (Gallego Romero et al., 2014). As the samples had different slaughter days, which affected the collection conditions, we also deemed it necessary to correct for this via the batch effect. Finally, we corrected for breed and age at slaughter, as these are important biological factors, which could cause differences in expression.

We used the following three methods for the differential expression analysis (DEA): Limma (Ritchie et al., 2015), edgeR (Robinson et al., 2010), and Deseq2 (Love et al., 2014). This was done to increase the robustness of our analysis, as our phenotype of interest is expected to have a subtle effect on the transcriptome due to the complex nature of FE. In addition, we also fit the model for each breed separately using Deseq2, just removing the Breed as a covariate.

#### Deseq2

We used Deseq2 version 1.22.2. In the Deseq2 analysis, the counts were filtered *a priori* requiring a minimum of 5 reads for each sample, resulting in a total of 10765 out of 25880 genes being included in the DE analysis in the joint breed analysis, and 10687 and 11107 in Landrace and Duroc respectively. As the overall read counts were very similar across experiments (see Supplementary Data 1), it was deemed sufficient to filter pre normalizing. We then used the default analysis method based on our specified model.

#### Limma

We used Limma version 3.38.3. For the Limma analysis, the counts were filtered based on the edgeR *filterByExpr*function and normalized using *calcNormFactors* from the same package, as suggested in the limma manual. This filtering was done on the full data including both breeds. This resulted in the inclusion of 11146 genes in the analysis. To fit the model we used the *eBayes* method in conjunction with our specified model.

#### EdgeR

We used edgeR 3.24.3. We used the same normalization and filtering as in the Limma analysis, thus including the same number of genes. We used the *glmQLfit* function and *glmQLTest* to implement our model.

While we used two different gene set sizes in the analysis, this did not affect the results significantly, as the genes omitted in the Deseq2 analysis were all lowly expressed. Furthermore, in our further analysis we elected to use the smaller and more conservative Deseq2 gene set to become our reference set for further selections and analysis. In total, 99.9% of genes in the Deseq2 gene set were also in the Limma/edgeR set.

### Gene Pathway Analysis

#### Gene Selection

To select a robust set of genes for a gene enrichment analysis when we have non-conservative *p*-value but only a limited number of genes with a FDR below 0.05, we applied the following strategy:

– Identify the overrepresentation of (low) *p*-values in comparison to a uniform p-value distribution in our data. We will call this the divergent count.
– Select the top N genes by *p*-value, where N is the estimated divergent count.
– Among the top N genes, select those that are found in all three methods.

To find the divergent count D, we find the interval with the maximum positive divergence between our observed empirical *p*-values and the same number of uniformly distributed *p*-values. This is completely analogous to the KS-test with the uniform distribution from 0 to 1 as a reference, and thus the probability of a given divergence is simply the KS-test *p*-value between our empirical data and the theoretical uniform distribution. It is calculated as follows:

(1) $$d_{i}=(\sum_{n}^{i=1}p_{i}\left\{\begin{array}{cc} 0forx_{i}>\frac{i}{n}\; & \\ 1forx_{i}\leq \frac{i}{n}\; & \end{array}\right)-i$$

(2) $$D=\text{max}\left\{d_{1},d_{2}\ldots d_{n}\right\}$$

Where n is the total number of *p*-values, *p*_*i*_ is the i’th observed *p*-value in increasing order. Here, i is both the index for x and the expected number of *p*-values between 0 and $\frac{i}{n}$ given a uniform distribution. Note that to get the actual KS-test metric, *d*_*i*_ and i are divided with n. D is the final divergent count, which is the maximum over all possible values of *d*. This represents the excess number of low *p*-values, given the following assumptions:

1. The *p*-value mass distribution is approximately decreasing toward lower p-values.
2. The divergence should be significant.

Once the maximum divergence is found and the assumptions are fulfilled, the next step is to assign a probability to this divergence. As mentioned above, this is simply the KS-test between the observed *p*-values and the uniform reference distribution.

##### GOrilla

To perform gene enrichment in GOrilla (Eden et al., 2007, 2009), we translated our *Sus scrofra* ensemble gene IDs into human ensemble gene IDs. The background set of genes used in GOrilla was the set of genes from the Deseq2 analysis. We used default settings. Furthermore, we used the Revigo (Supek et al., 2011) analysis through GOrilla to generate summaries of our enrichment analysis, using default settings.

#### Feed Efficiency Measure

In this study, we elected to use weight gain/feed intake as our FCR measure. It fit the data better than RFI, and FCR is the metric used in the Danish breeding program.

### Pairwise Gene Interaction Analysis

To continue our analysis of the top set of genes identified using the divergent counts in our DE analysis, we decided to apply a pairwise interaction model. First, we adjusted the expression based on any factors and covariates that may affect expression for each gene. These factors are the same as in the general DE analysis, giving rise to the following linear model:

$$y_{jklm}=\mu +\beta_{1_{j}}\left(RIN\right)+\beta_{2_{k}}\left(age\right)+BR_{l}+BA_{m}+\epsilon$$

y = normalized read counts

β_1_ = *regression coefficient of RIN (RNA Integrity value)*

β_2_ = *regression coefficient of Slaugter Age (days)*

BR = Breed

BA = Batch

We then centered and scaled the residuals and then run a model for all pairwise gene interaction in our gene set We scaled and centered because this leads to a more flexible and interpretable model regardless of the type of interaction. The interaction model was as follows:

$$y_{i}=\mu +\beta_{1}x_{1_{j}}+\beta_{2}x_{2_{k}}+\beta_{3}\left(x_{1_{j}}\times x_{2_{k}}\right)+\epsilon$$

y=FCR values

β_1_ = *regression coefficient of residual expression of gene 1*

β_2_ = *regression coefficient of residual expression of gene 1*

β_3_ = *regression coefficient of the interaction between gene 1 and gene 2 *x*_1_*j*__ = residual expression of gene 1*

*x*_2_*k*__ = residual expression of gene 2

(*x*_1_*j*__×*x*_2_*k*__) = product of the two residual expression values

The next step was then to identify significant interactions. As the number of interactions in a dataset grows exponentially to the square of the input space, it is often difficult to detect effects based on classical multiple testing correction methods such as Bonferroni or FDR. This is especially true when dealing with complex phenotypes, as we generally do not expect to find individual large effects. Therefore, instead of focusing on individual results for each gene, we calculated the divergent count, to assess the divergence of each genes’ distribution of interaction *p*-values. We then bootstrapped with replacement samples of 853 *p*-values from our empirical *p*-values 10^5^ times, calculating the divergent count each time, giving us a bootstrapped distribution of divergent counts, to compare with our empirical distribution.

### Weighted Gene Network Analysis

To perform network analysis, we used weighted gene correlation network analysis (WGCNA) (Langfelder and Horvath, 2008). First, we filtered the read counts to only include genes with a minimum of 5 un-normalized reads, as was done for the Deseq2 analysis. We then created a correlation matrix based on all pairwise correlation in the data. We calculated the correlation matrix based on uncorrected expression values, as the individual gene-gene pairwise correlation are based on within-pig comparisons. We then fit the ß parameter for the scaling of the network to create a scale free topology (Zhang and Horvath, 2005). The ß scaled correlation matrix was our adjacency matrix, which was used to generate the Topological Overlap Measures (TOM), which represents the final calculation of the relation between genes.

The TOM values of the genes where clustered using the *dynamicTreeCut* function from the dynamicTreeCut cut package with default setting, resulting in a number of modules arbitrarily differentiated based on colors. The eigenvalue of each module was then calculated based on the normalized read counts and RIN adjusted count. We did these corrections in this step to remove the technical effects of library size differences and RIN from the eigenvalues, as we did not want technical effects to affect the eigenvalues. The counts were normalized based on the *calcNormFactors* function from the edgeR package. After this, the counts were adjusted for RIN by fitting the following linear model: *expression* = μ + RIN + *ϵ* for all genes, and extracting the residual expression values. Highly correlating models where merged using the *mergeCloseModules* function using a default cut-off. We then calculated the Pearson correlation between corrected and normalized module eigenvalues and our traits and covariates. Pathway analysis was performed on the genes of highly correlated modules, with GOrilla and ReviGO as seen above. Finally, we also identified the top hub genes in the high correlation modules. This was done based on calculating the intramodular connectivity using the *intramodularConnectivity* function with default settings. We then selected the top hub genes base on the k within measure, which represents the connectivity within modules.

### Comparison to Human Exercise Data

To test the hypothesis that differences in the muscle tissue transcriptome of Duroc and Landrace and/or FCR related genes mimic differences in rested and exercised muscle tissue, we compared our results with three human data sets, all analyzing the leg muscle transcriptome during exercise (Murton et al., 2014; Devarshi et al., 2018; Popov et al., 2019). The Murton et al. (2014) dataset was from an analysis of the time series analysis of transcriptome changes based on resistance training in leg muscle in 8 untrained men. The Popov et al. data came from an analysis of acute changes in the leg muscle transcriptome after endurance training, with 7 male subjects. Finally, the Devarshi et al. (2018) dataset was an analysis of the effect of acute aerobic exercise on the leg muscle transcriptome in lean and obese men, with a total of 30 subjects. For each data set, we performed the following:

1. We selected the genes differentially expressed between breeds, based on the edgeR analysis.
2. For FCR, we used the 853 genes from divergent count set.
3. We found the same set of genes in the human data – the breed/FCR matching genes. Genes were matched using the biomart R package, based on retrieving the external_gene_name of our Sus scrofa ensemble gene identifiers.
4. We separated the human data into two parts – the breed matching set and the background set. The breed matching set is the set of genes which were differentially expressed by breed, at 0.05 FDR in between breeds, which were then matched to the human genes by translating the gene identifiers between species. The background set were the remaining human genes.
5. We applied the Fisher Exact test to compare the number of differentially expressed genes for the exercised vs. rested muscle in the background set vs. the breed matching set.
6. The steps for the breed were also applied to our divergent count set for FCR.
7. Pathway analysis using GOrilla was performed in both the breed and FCR gene sets. The genes used were the intersect between all the DE genes from the human studies and the breed and FCR sets, respectively.

In this part of the analysis edgeR was used because it was more flexible to fit to the publicly available data, allowing us to compare our results to the other studies. As each dataset was formatted and analyzed differently, we had to process them individually. In the data set from Devarshi et al. (2018) (dataset 1), we chose to use the lean pre exercise vs. lean post-exercise group as our comparison, and significance was based on the reported cuffdiff analysis. For the set of Murton et al. (2014) (dataset 2), we pooled all control vs. exercise samples and analyzed them using Limma as the data was microarray data, using the same Limma pipeline as mentioned above in our FE analysis. As the results were weaker in Murton et al. (2014), we chose to use *P* < 0.05 as a cutoff for the Fisher exact test. For the set from Popov et al. (2019) (dataset 3), we grouped all the 4 h post-exercise results vs. all 4 h control non-exercised and performed DE analysis using edgeR with no other covariates using the same settings as our FE analysis above, with significance based on the found FDR values.

## Results

### Differential Expression Analysis

**FIGURE 1:**
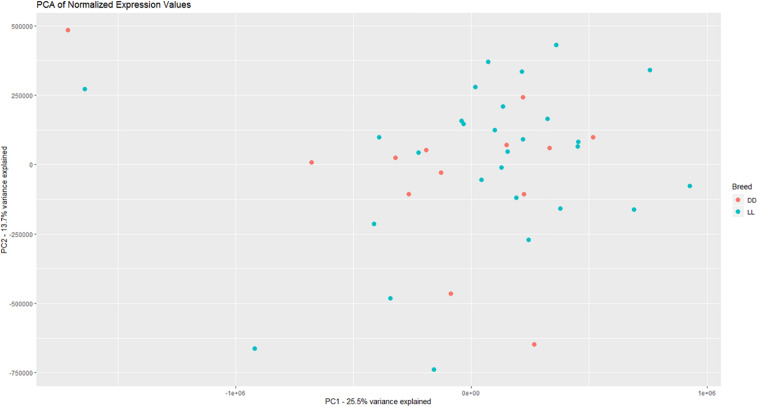
Visualization of the two first principle components in the expression data, with DD being Duroc and LL being Landrace. There is not a clear separation between breeds based on the overall expression, giving credence to a joint breed analysis of the data.

**FIGURE 2:**
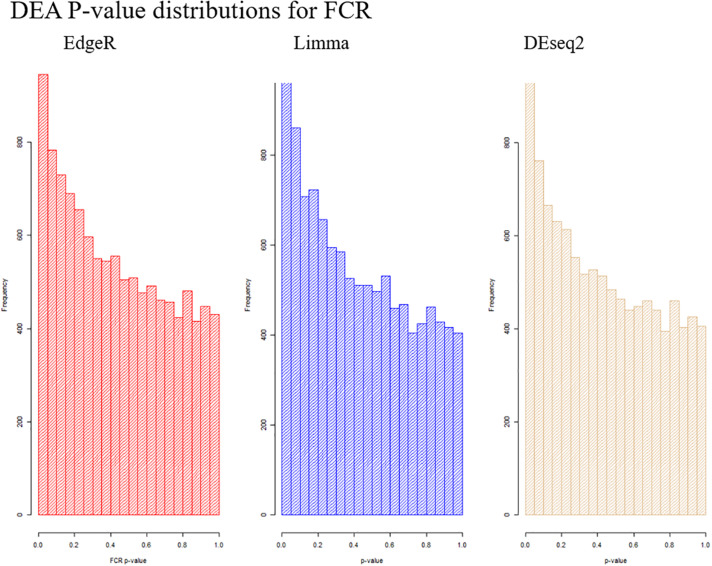
Visualization of the distribution of the *p*-values testing the relation between FCR and gene expression for all three analysis methods. It is clear in all cases that we observe an anti-conservative distribution, that is, there is an overweight of low *p*-values.

Based on PCA, there is no clear separation between the two breeds based on the first two components (Figure 1). This confirms the relevance of a joint breed analysis. It is still possible that principal components beyond the two first are well correlated with breed. However, as lower components will explain less of the overall variation, the majority of the variation cannot be explained by breed alone. Naturally, this does not mean individual genes do not have different expression due to breed, as we see in the DEA. In the Deseq2 DEA, the Landrace analysis had one gene with an FDR < 0.1, the Duroc analysis had 8, and we found 4 in the joint breed analysis (Table 1). This is quite low numbers in comparison with the rest of the covariates (Table 1). When we viewed the overall *p*-value distribution for FCR in the Deseq2 DEA, we found that the Duroc distribution was slightly skewed toward high *p*-values (Supplementary Figure S1 right), the Landrace distribution had a slight excess of high *p*-values (Supplementary Figure S1 left), but the joint analysis had a clear excess of low *p*-values (Figure 2 right). As the results for FCR were somewhat limited, we chose to continue with a different strategy based on the joint breed analysis. We chose to calculate the DE using 3 methods, ensuring that the results were robust and replicable, as individual methods can vary in output (Seyednasrollah et al., 2015). Observing the distribution of uncorrected *p*-values for FCR in all 3 methods (Figure 2), we found an anti-conservative distribution regardless of the method. If FCR was unrelated to gene expression in general, we would expect a uniform *p*-value distribution in our model. To test the likelihood of the observed results being generated by a uniform distribution, we applied the KS-test, comparing the empirical values with a theoretical uniform *p*-value distribution. The results showed that it was very statistically unlikely that the *p*-values had an underlying uniform distribution for all three DE methods (*p* < 10^–16^). This lead us to conclude that there was a relation between the muscle tissue expression and FCR. Overall, the most significant covariate was RIN (Table 2), highlighting the importance of correcting for the RIN values when analyzing samples acquired in a non-laboratory setting. It has been previously shown that RIN has an impact on expression values, but explicitly controlling for this in a modeling framework should appropriately correct the data (Gallego Romero et al., 2014). Furthermore, many genes were differentially expressed between the breeds and due to age differences. To quantify the observed link between expression and FCR, we continued with two strategies – analyzing the overall pathway enrichments for the most significant genes and creating gene expression modules based on network analysis of the gene expression profiles.

**TABLE 1:** Overview of genes with a FDR value < 0.1 in all 3 differential expression analysis.

Gene name	Breed	FDR	Regulation
PNCK	Landrace	0.0007	Down
Patr-A	Landrace	0.08	Down
MTMR11	Duroc	0.07	Up
C3	Duroc	0.02	Down
LCP1	Duroc	0.02	Up
TRIM63	Duroc	0.08	Down
KLHL30	Duroc	0.07	Down
NANOS1	Duroc	0.08	Up
IGHM	Duroc	0.07	Up
ETV5	Duroc	0.02	Down
MTFR1	Both	0.068	Down
MGAT4A	Both	0.098	Down
SLC38A2	Both	0.098	Up
MRPS11	Both	0.067	Up

*There is only a limited amount of genes differentially expressed at 0.1 FDR level for FE. Notably, out of 4 genes in the joint breed analysis there are two genes with mitochondrial related Gene Ontologies – MRPS11, MTRM1. MTFR1 has been implicated in eating quality (measures of meat quality post-cooking) in cattle (Jiang et al., 2009) and as a meat PH QTL in pig (Chung et al., 2015). Also interesting to note that TRIM63 has been suggested as a biomarker for difference in response to exercise-induced muscle damage (Baumert et al., 2018), KLHL30 has been associated with intramuscular fat and muscle metabolism in Nelore Cattle (Dos Santos Silva et al., 2019). MGAT4A has been linked to diabetes and glucose transport (Ohtsubo et al., 2005).*

**TABLE 2:** Overview over the number of genes with FDR < 0.1 in the joint breed analysis for all 3 methods and each covariate.

Trait	Deseq2	Limma	EdgeR
FCR	4	0	0
Breed	3633	3679	3428
RIN	5572	5763	5779
Age	503	189	328

*In general, we have modest amount of DE genes for FE, while our other covariates have many significant genes associated with them.*

### Enrichments Analysis

**FIGURE 3:**
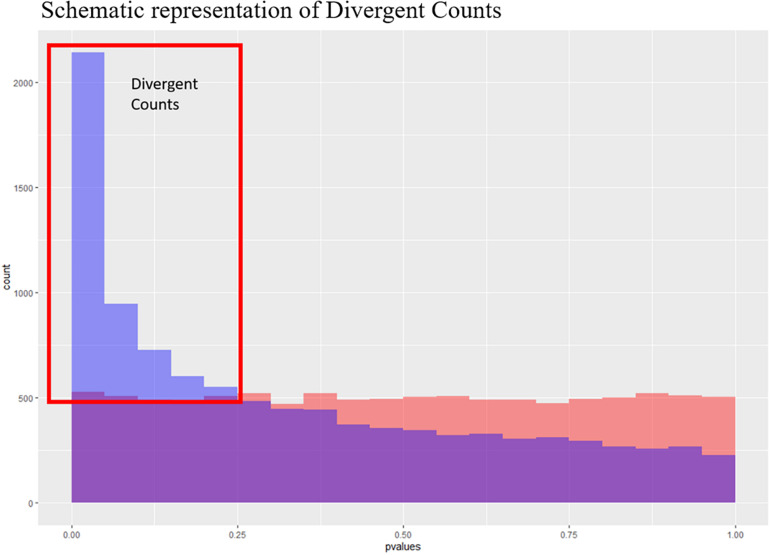
Schematic representation of the divergent counts. Here we see to theoretical *p*-value distributions, one which is uniform (in red) and one which is anti-conservative (blue). The purple area is where they overlap, and the blue area is the area used to estimate the divergent counts.

**FIGURE 4:**
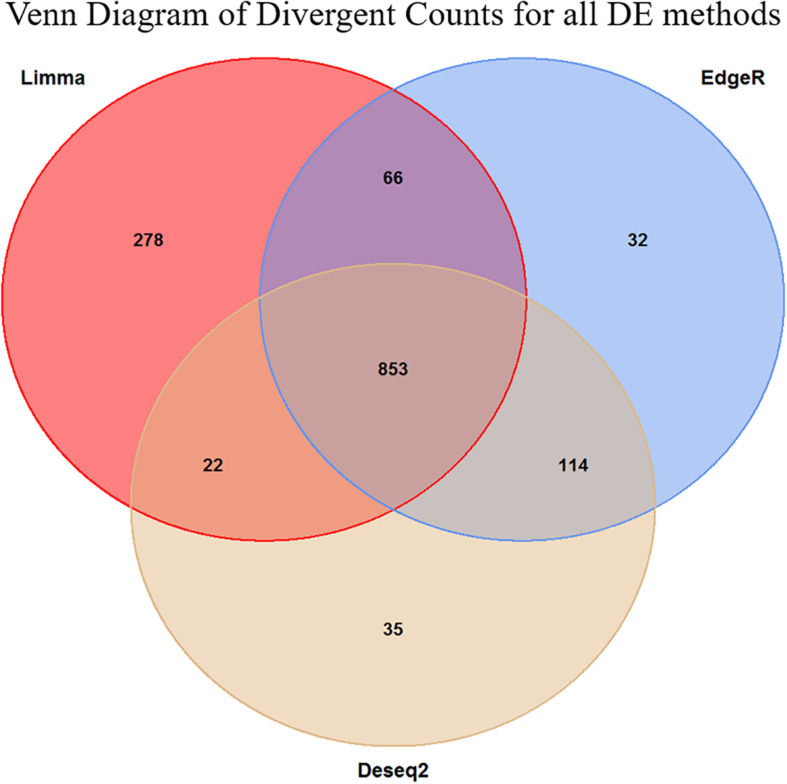
Venn diagram of the overlap in the divergent counts between the three methods. We see here that the Limma is overall less conservative than the two other methods, but in general, the methods are in high agreement with each other. The final set of genes selected for the enrichment analysis was the 853 triple overlapping set.

**FIGURE 5:**
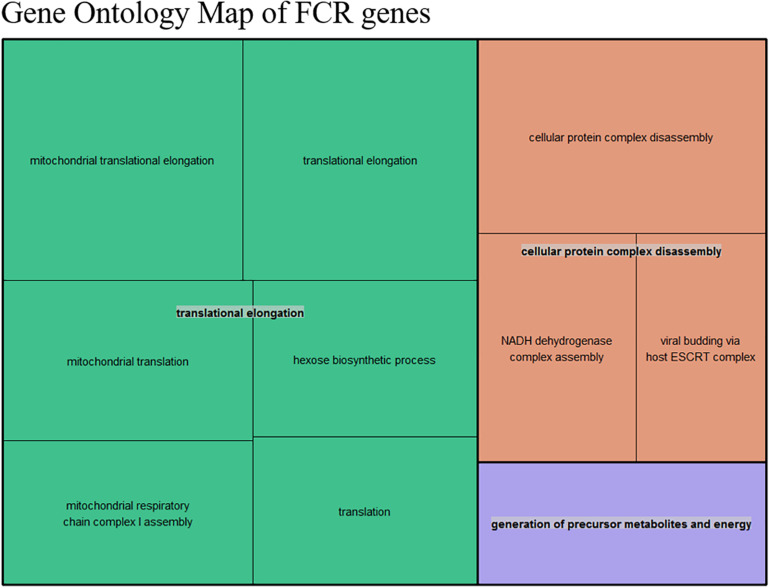
Summarized representation of significant GO- for the genes set generated from the divergent count (853 total genes) overlap based from the DE analysis of FCR. The size of the boxes is scaled according to the −log10 of the *p*-value. The most significant individual terms are all in the translation, indicating a link between mitochondrial activity and FE.

The first step in an enrichment analysis is to select a suitable set of genes. The most general strategy is to pick genes that are differentially expressed after multiple testing correction for such a set. Based on the DE results, we did not have enough of DE genes for selecting a meaningful gene set for enrichment analysis, but we were able to demonstrate an overall relation between FCR and gene expression (Figure 2). One could select genes with an uncorrected *p*-value below 0.05 for pathway enrichment, but this is somewhat arbitrary selection (Butler and Jones, 2018). Instead, we made an estimation of the number of surplus low *p*-values in comparison to uniformly distributed *p*-values. The uniform *p*-values represented the null hypothesis of no overall relation between FCR and gene expression. We called this value the divergent count. In essence, we estimated the interval with the maximum positive divergence between our observed *p*-value frequencies and the same number of uniformly distributed *p*-values, assuming an approximately monotonely decreasing *p*-value distribution in our results (Figure 3). This had the advantage of not relying on arbitrary cutoffs, instead giving a set of genes proportional to the overall divergence of the *p*-value distribution. As the divergence calculated was analogous to the test metric of the KS-test, the probability of observing the empirical observed divergence was given by our KS-test above (*p* < 10^–16^). Based on the overlap of the genes selected between the 3 DE methods, the majority of the selected genes are identified by all three methods (Figure 4). This indicates that the final gene set for pathway enrichment was robust. To identify enriched functional pathways in the final overlapping gene set, we used GOrilla (Eden et al., 2009). In GOrilla, it is possible to give a background set to base the analysis on, making it advantageous for expression data, as it allows us to use genes expressed in our data as a background. We identified, 5 terms as significant post-multiple corrections, with 4 out of these being related to mitochondrial ontologies (Supplementary Data 2). A summarized output of the significant GO terms after multiple testing correction based on the GOrilla analysis, using Revigo (Supek et al., 2011) revealed translation elongation as the main overall grouping of the terms (Figure 5).

### Gene-to-Gene Expression Interaction Analysis

Many strategies can be used to take advantage of the interaction or co-expression between genes. We applied modeling of pairwise gene interactions explicitly including the phenotype as an outcome variable in the model. This can be advantageous when dealing with complex phenotypes, as it may make it possible to capture subtle biological variation. We performed the gene interaction analysis based on the set of genes we identified from the overlap of all 3 methods from the DE analysis, based on the calculated divergent count for each method. When comparing the empirical values with the bootstrapped values the maximum bootstrapped divergent count was 83, while there were 193 genes with a divergent count over 83 in the empirical data. As the bootstrapping was based on 10^5^ samplings, it verifies that the empirically observed interactions are quite unlikely to be random effects. One caveat is that as this analysis was based on genes pairs, the divergent counts of each gene were not independent from the values of other genes. Due to the issue of independence and general concern of data size and weak effects we used a conservative qualitative heuristic and focused on the top 20 genes based on our methodology in the discussion.

### Weighted Gene Network Analysis

**FIGURE 6:**
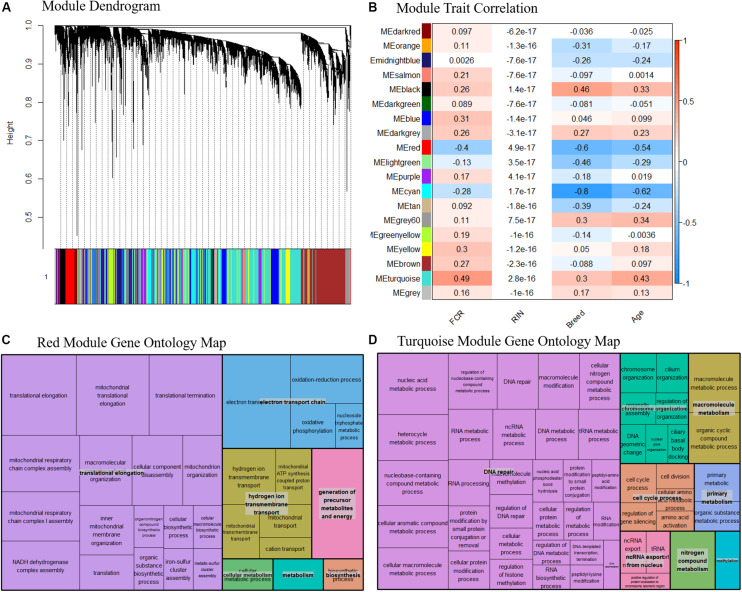
**(A)** Dendrogram over the module clustering. Looking at the visual clustering not all the modules look equally well defined, but it should be noted that the actual relations in given module cannot be simplified to two dimensions, as the all the relations between the genes exist in N dimensional space, where N is the number of genes. **(B)** Correlation between module eigenvalue and our traits, including RIN. We see here that the correlation to RIN is essentially 0 in all cases, indicating our linear correction method has worked well. Based on the top two modules **(C)** Summarized representation of significant GO- for genes in the red module of the WGCNA network analysis. The three largest groups are all associated with mitochondria, mirroring the results found in the differential expression analysis and the gene interaction analysis. **(D)** Summarized representation of significant GO- for genes in the turquoise module of the WGCNA network analysis. The main grouping here is DNA repair, which is not found in our other analysis. This may represent that increased energy expenditure on maintenance processes is reducing FE.

Based on our network analysis, we identified 19 distinct modules after correcting for RIN and merging the modules based on similarity. Due to the initial DE results, we decided not to focus individually on Landrace or Duroc pigs in the network analysis, and thus the network was generated combining both breeds. The hierarchical clustering of the modules might give the impression that the network is poorly constructed, as the module dendrogram representation is not very clear (Figure 6A). In general, some modules were tightly clustered based on the dendrogram, such as the red module, while others seemed more diffuse. One should realize however, that the modules themselves were based on N × N matrix, where N is > 10^5^. Thus, the dendrogram could only act as a visual guide, and not show the full picture. Therefore, we relied on the correlation between module eigenvalues and traits combined with pathway analysis of the modules to assess if the modules were biologically meaningful. The effect of the removal of the effect of RIN on a gene by gene basis effectively removed any correlation between RIN and the eigenvalues of our modules (Figure 6B). Several of the modules were well correlated with the breed and age, with correlation > 0.5, while FCR was mainly correlated with two modules, the red and turquoise (Figure 6B). The red and turquoise modules included 391 and 3744 genes, respectively. The red module was more correlated to breed and age than FCR, but previous knowledge indicated that breed and FCR are generally correlated between Durocs and Landrace, as Durocs are more efficient. Furthermore, age was correlated with FCR (0.5) in the sampled pigs. It should, however, be noted that the age-FCR correlation in the pigs was created due to the ending of the feeding trail being based of a fixed weight of 100 kg. Thus, the lower FCR pigs took longer time to reach 100 kg, and had a higher slaughter age and tissue sampling age. This means that the correlation was not due to underlying biological effects. The turquoise module showed highest correlations with FCR (Figure 6B). We performed pathway analysis using GOrilla and Revigo on the genes in the red and turquoise modules (Figures 6C,D, respectively). In both the red and turquoise modules, a large number of GO terms were significantly overrepresented after multiple testing correction (see Supplementary Data 4, 5 for the full list of red and turquoise GO-terms, respectively), indicating that the modules represented specific biological pathways. In the red module, the most significant group of terms was related to mitochondria. These terms were grouped into three overall groups – translation elongation, electron transport chain and hydrogen ion transmembrane transport (Figure 6C). This mirrors our finding from the DE analysis and the gene interaction analysis. As the module had a negative correlation with FCR, indicating a relation between higher mitochondrial activity and lower FCR, thus higher efficiency. In the turquoise module, there was one large grouping of terms – DNA repair. This category included many GO terms, related to RNA, DNA, amino acid and nucleic acid metabolism and processing (Figure 6B). We also calculated the top 10 genes in terms of module connectivity in the red and turquoise modules (Supplementary Data 6). Interestingly, in the red module, 7 out of 10 genes belonged to the NADH ubiquinone oxidoreductase group (NDUF), with the remaining 3 also being implicated in mitochondrial function. Thus, the mitochondrial genes were both overrepresented in the red module and the most connected within the module. In the turquoise module, the results were unclear, as the most connected genes did not belong to any specific process, but instead covered a range of general processes that are important for cell function. This fits well with the large size of the module and the overrepresented GO terms found.

### Human Exercise Data

**FIGURE 7:**
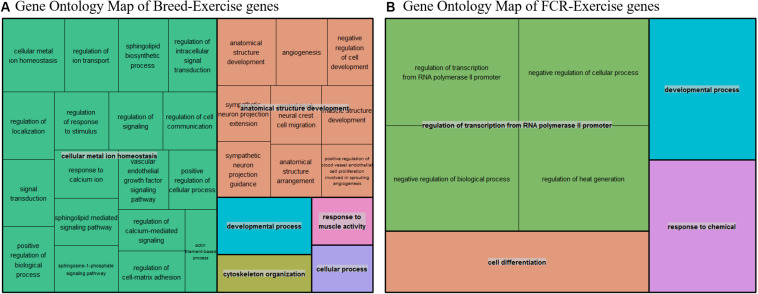
**(A)** Summarized representation of significant GO- for genes significantly associated with exercise in one of the three human dataset and between the breeds, based on a total of 702 genes. The size of the boxes is scaled according to the −log10 of the *p*-value. Here we find two overall main categories, cellular metal ion homeostasis and anatomical structure development. **(B)** Summarized representation of significant GO- for genes significantly associated with exercise in one of the three human dataset and in our divergent set for FCR. The size of the boxes is scaled according to the −log10 of the *p*-value. Here the main process is regulation of transcription from RNA polymerase. Overall, the categories are not very significant here as it is only based on 42 genes.

To test the hypothesis that improvements in efficiency could be linked to a state mimicking exercise, we compared our divergent counts genes for FCR and the genes differentially expressed between breeds with three different human exercise datasets [33–35]. We compared if there was a higher proportion of genes that were significant for exercise-mediated changes in the subset of genes which were identified based on differences in breed and FCR, in relation to the remaining background set of genes remaining genes. For all 3 datasets there was a higher proportion of significant genes in the breed and FCR sets versus the background set, as the odds ratio between the subsets and the background was always below one (Table 3). In general, the results for the breed related genes were more significant than for the FCR genes, but they showed similar ratios. This is likely because there were roughly 4 times more breed genes, yielding higher statistical power. The results did give some confirmation to the hypothesis that the FCR and breed related genes were more significant than for exercise related changes than the background genes. We also did pathway enrichment analysis for the genes that were significant in any of the human data sets and in the breed, and FCR set respectively (Figures 7A,B). In the human-breed overlap genes, the main categories were cellular metal ion homeostasis and anatomical structure development, based on 702 genes. For FCR, only 42 genes overlapped with the significant human genes. This means the overall pool of genes was too small for significant enrichment, but the main pathway identified was regulation of transcription from RNA polymerase II promoter.

**TABLE 3:** Results of Fisher exact test comparing the number of genes significant for difference in rested and exercised muscle in divergent count genes for genes found in the divergent count for FCR and breed and the background for each of the 3 human data sets [dataset 1 (Devarshi et al., 2018), dataset 2 (Murton et al., 2014), and dataset 3 (Popov et al., 2019)].

Data	*P*-value breed	Odds ratio breed	*P*-value FCR	Odds ratio FCR
Dataset 1	0.0017	0.79	0.0046	0.71
Dataset 2	0.0012	0.85	0.22	0.9
Dataset 3	0.12	0.84	0.47	0.88

## Discussion

There have been four previous studies analyzing the muscle transcriptome in an FE context (Jing et al., 2015; Vincent et al., 2015; Gondret et al., 2017; Horodyska et al., 2018b).

The study by Gondret et al. was based on selecting divergent FE lines of Large White pigs for 8 generations. It included a total of 24 pigs and was based on microarrays. They reported a high number of differentially expressed genes between the low and high RFI groups (2417), but it is not clear from their paper how many probes were included in the statistical analysis and how this may have affected multiple testing correction. They also reported that a gene was considered differentially expressed if one probe met the cutoff regardless of the amount of probes in a given gene. They reported mitochondrial electron chain transport, glucose metabolic process and generation of precursor metabolites and energy as pathways significantly associated with RFI.

The study from Horodyska et al. used 16 pigs, but included 8 pigs of each gender. They used an uncorrected *p*-value of 0.01 as their significance threshold for DE, without properly motivating this decision. They reported 272 genes with *p* < 0.01, which is similar to what we found in the DESeq2 analysis (243 genes with *p* < 0.01). However, we have included less genes in the analysis (14497 vs. 10563).

Vincent et al. (2015) included 16 female Large Whites from divergent RFI lines. Their study was based on microarray. They reported their results based on uncorrected *p*-values in both expression and proteomics. As in our study, they found mitochondrial related probes being significantly associated to RFI.

Finally, in Jing et al., a total of 6 Yorkshire pigs were used, based on the selection of high and low extreme values for RFI values in 236. They reported 645 DE genes, with 99 genes having a reported FDR lower than 0.05. However, selecting such few samples at the extreme end of FE does raise the question of replication, as the large differences in RFI/FCR they achieved could easily be caused by factors that are not generally applicable, such as underlying disease. Indeed, it is not realistic to observe such large differences in FE based solely on genetics in a production population, based on the distribution we find in our data. They found that the most significant pathways in their data were mitochondrial activity, glycolysis and the myogenesis pathways.

If we compare our study with the previously reported studies, we have the highest number of samples reported (41) and we included two breeds, which none of the other studies did. In contrast to other studies, we did not have any divergent selection for FE, but the Duroc pigs in this study have been more strongly selected for FCR than the Landrace pigs, giving us a level of divergence based on real breeding goals and current pig industry practices. Having this setup does present advantages and disadvantages. The advantage in relation to the other studies is that the results may generalize better across breeds. The disadvantage is that we may have fitted breed effects instead of phenotypic effects, but breed is accounted for in all the performed analysis. The other main difference is that we have fitted FCR as a continuous value. In general fitting a continuous value should be more applicable to pig production. In breeding populations, FE is a continuous variable, and so are breeding values. When breeding values are predicted, they are assumed to be a sum of additive effects, and not a binary categorization. Beyond this, in pig production, there is no low FE selected line to contrast with, so while the studies using divergent lines may identify biological factors that affect FE, these may not be relevant to non-divergent populations. Despite the issues presented with these four previously conducted studies, it is notable that mitochondria are reported to be related to FE multiple times, as well as in this study. Another general issue which arose, is how to deal with statistical issues in analysis of FE. From the various studies presented above it is clear that the connection between FE and the muscle transcriptome is subtle. Here, we tried to tackle this issue by not being overly conservative, but still applying multiple testing correction using a FDR of 0.1 level for individual results in our DE analysis. Furthermore, we relied on the overall distribution of results and/or combination of genes in groups, to avoid relying on individual weak effects.

### Differential Expression Analysis and Pathway Enrichment

In the DEA, we identified 14 genes with an FDR value below 0.1. Of these 14, six genes had been associated with production traits or other functionality that could be relevant in an FE context. As in previous studies, we found genes related to mitochondria (MRPS11, MTRM1). We also identified a gene associated with glucose metabolism (MGAT4A) (Ohtsubo et al., 2005). Two genes were associated with meat quality phenotypes in cattle and pig (MTRF1, KLH30) (Jiang et al., 2009; Chung et al., 2015; Dos Santos Silva et al., 2019). Perhaps the most interesting result, is that one of the genes found in the Duroc analysis, TRIM63, has been associated as a biomarker for differences in response to exercise induced muscle damage (Baumert et al., 2018), which ties into our comparison to human data. No general conclusions about the general pathways involved in FCR could be made, given the low amount of DE genes.

Instead, we chose to use a novel approach for selecting an expanded set of genes to make a pathway analysis possible. First, to make the analysis more robust, we choose to base the pathway analysis on results from three DE expression methods. Furthermore, we chose to select genes based on the overall divergence from the null hypothesis of our *p*-value distribution, as this should represent a set of genes that was likely to be associated with our trait, even if the genes were not significant based on individual FDR corrected *p*-values. To our knowledge, this was a novel way of selecting a group of genes, which we called the divergent count. This method was motivated and based on two important factors. First, we required a left-skewed *p*-value distribution, which should be approximately monotonely decreasing, which was the empirical distribution of our *p*-values (Figure 2). Second, the divergence must be significant. Due to the way we calculated our divergence, the probability of a given divergence is well understood, and is simply the KS-test of the *p*-values. The enriched pathways in our dataset selected based on the divergent counts revealed that all significantly enriched pathways were associated with mitochondrial genes (Supplementary Data 2). The association of mitochondrial activity and FE has been found in several species beyond the pig studies already mentioned above, such as cattle and broiler chicken (Connor et al., 2010; Bottje et al., 2017). While this is not a novel result, we found it in a novel setting, with a larger sample size, a novel population and using a continuous value for FCR. This acts as further evidence to the link of mitochondrial activity and FE, but also as evidence that it may be relevant in real breeding populations, and not only in divergently selected test populations. Finally, it also gives us some biological confirmation of the genes selected by the divergent count, due to the confirmation of previous results.

### Gene-to-Gene Expression Interaction

The gene expression interaction analysis was a novel way of finding genes with a high degree of interaction with other genes in relation to a trait of interest, which had not been applied to FE in pigs before. According to the way we modeled the effects and selected the top genes identified were the genes that had most significant interactions effects with other genes in relation to changes in FCR. From the top 20 genes (Supplementary Data 3), the most interesting genes based on previous literature and function were: several transcription regulators: ETV1 (an androgen receptor activated gene), LF1 (transcription factor) and KDM4C (transcription activator and growth related gene) (Bray and Kafatos, 1991; Cai et al., 2007; Gregory and Cheung, 2014); two mitochondrial genes, KMO and MRPS11 (Meinke et al., 2019); two genes related to muscular atrophy – GEMIN7 and PLPP7 (Baccon et al., 2002; Meinke et al., 2019); one gene implicated in heart development BIN1 (Nicot et al., 2007), two lipid metabolism/obesity related genes ACOT11 and GPD1 (Adams et al., 2001; Park et al., 2006); and finally 3 genes associated with specific traits in pig IL2RG (Immune system in pigs) (Suzuki et al., 2012), GGPS1 (meat quality) and PPARA (weak association with fat percentage) (Szczerbal et al., 2007). Interestingly, MRPS11 was also differentially expressed in the DEA. How should one interpret such a mixed set of functional results? Given the way these genes were identified, we did necessarily expect them to be from a single pathway, but we would expect them to have functions that would allow for significant interaction with many genes, while being relevant to FCR. Thus, transcription factors, genes involved in energy metabolism and muscle development all qualitatively fit genes that could have an important role in FE related processes. Finally, we also found mitochondrial genes in the interaction analysis, giving further evidence to the link between mitochondria and FE.

### Gene Network Analysis

The gene network analysis revealed that the red and turquoise modules were the only modules with a correlation > 0.4 with FCR. Based on the GO term analysis enrichment of the red module, we found many enriched GO terms related to mitochondrial processes, confirming our finding the DEA and network analysis, and from other studies (Connor et al., 2010; Jing et al., 2015; Vincent et al., 2015; Bottje et al., 2017; Gondret et al., 2017). More specifically, the negative correlation between the red module eigenvalue and FCR also showed that higher mitochondrial activity was positively associated with higher efficiency. This was further confirmed, as the top ten most connected genes in the module were all related to mitochondria. Interestingly, seven of the top ten genes were from the NDUF family, making this gene family into an interesting candidate for future study and biomarker development. The turquoise module was the module with the highest overall correlation (0.49). Furthermore, it was more correlated to FCR than traits, meaning the correlation was less likely to be driven by collinearity with the other traits. Based on the GO term analysis, we found that the module was highly enriched for genes related to DNA repair, which included GO terms related to RNA, DNA, amino acid and nucleic acid metabolism and processing. To the best of our knowledge, this is the first evidence of these processes being related to FE in general. The only previous link to DNA repair in livestock was a feed restriction study of cattle (Connor et al., 2010). These processes could be generic growth and maintenance processes, and as the module is positively correlated with FCR, we can speculate that higher activity in DNA repair and related processes are increasing energy expenditure on maintenance, thus lowering efficiency. The large number of genes in the module somewhat confirms the general metabolism and maintenance theory, as it is unlikely that very specific functional pathways should cluster together to form a large cluster. Further evidence to this was that the top ten hub genes of this module did not belong to a single specific pathway as in the red module, with the genes being involved in a wide range of processes related to general cell maintenance.

### The Mitochondrial Link

How does the ubiquitous link between mitochondria and FE functionally work? It does makes sense that an organelle which provides cellular energy will have an effect on the overall energetic efficiency of an animal. However, even though this link seems to well established, there are conflicting reports in the literature. Jing et al. (2015) and Vincent et al. (2015) report lower mitochondrial expression in more efficient pigs, while Bottje et al. (2017) and Gondret et al. (2017) report the opposite in pigs and broiler chicken. The down-regulation camp could argue that less mitochondria represent less energy spent. The effect of up-regulation in improving efficiency could be reduced oxidative stress and increased cell damage control (Bottje et al., 2017). The gene network analysis in this study pointed toward increased efficiency being related to higher expression. However, two DE mitochondrial genes from the joint breed analysis had opposite fold changes. Thus, while it is interesting that we confirmed the link between FE and mitochondria using pigs from an active breeding population, it is clear that a study specifically targeting the function of mitochondria and FE in pigs is necessary for explaining the exact functional background of this effect.

### Human Exercise

The overall functional background of FE in muscle tissue is still not very well established, despite some hints of mitochondrial effects. While there are a relatively small number of FE based muscle transcriptome studies, there are many studies analyzing other properties of the muscle transcriptome for other purposes. If it was possible to use previously published experiments as a tool for identifying functional aspects of FE, this could be a valuable resource that is relatively cheap to implement. This could generate and test novel hypothesis, and serve as a guide for further studies. As pigs as are commonly used as animal models for human disease, one could also do the reverse, and take advantage of human studies in the analysis of pig data. We hypothesized that differences between the Duroc and Landrace breeds, which have different overall FE, were more likely to be involved in processes related to exercise. The same hypothesis was also extended to genes related to FCR. This hypothesis was motivated by the fact that pigs are selected for lean growth. Exercise induced changes in muscle could thus be related to factors affecting lean growth in pigs. We found a slight confirmation of this hypothesis, as we found similar favorable odds ratio for our hypothesis in all three datasets, we tested for both FCR and our breed genes. The pathway enrichment analysis for the FCR and exercise related genes was not very statistically significant, as it only included genes. The main enriched category identified, based on four GO terms, was regulation of transcription from RNA polymerase II (pol II) promoters. Interestingly, Actin has been associated with the pre-initiation complex necessary for transcription by RNA polymerase II (Hofmann et al., 2004), which could be relevant given the importance of actin in muscle tissue (Tang, 2015). There are also links between a poll II subunit and myogenesis (Corbi et al., 2002). These results do provide relevant reasons for the observed enrichment, although more data is needed to confirm this due to the low number of genes used in the enrichment.

In the genes overlapping between exercise and breed differences, the results were more statistically robust, as they were based on a larger gene set of 702 genes. Here we found two main enrichment groups – cellular metal ion homeostasis and anatomical structure development. For the first term, we know that the transport of ions is generically vital to muscle function (Wolitzky and Fambrough, 1986; Mohr et al., 2007). The second term, anatomical structure development, is very generic in terms of function, and includes sub-categories that are related to muscle development, such as muscle structure development.

The results from the Human data analysis represented a novel hypothesis, but more analysis and new experiments on a larger population of pigs are necessary to strengthen the link between FE and exercise. One interesting aspect of this analysis is that pigs could be used as a model for lean growth in sedentary conditions, which could yield interesting therapeutic possibilities applicable to humans.

## Conclusion

Using multiple types of transcriptomic analysis based on novel biostatistical/bioinformatics methods (gene-to-gene expression interaction model, weighted network analyses, the divergent count method for gene selection and pathway enrichment using Kolmogorov-Smirnov test), we have reinforced the knowledge that mitochondrial activity is important for FE. The use of a non-divergently FE selected pig population reflects real pig industry practice that relies on naturally occurring genetic variation within breeds and populations for selective breeding. Based on the findings, we postulate that mitochondrial genes, and in particular genes from NDUF group or MRPS11 could be used as potential biomarkers for FCR in pigs and could be included in genomic selection program that distinguishes genomic regions. Furthermore, all our top genes from our interaction analysis also show promise as potential FCR biomarkers, that could be useful in selective pig breeding for FE. Finally, we found that there is a putative link between genes involved in exercise related changes in human, and FE in pigs, hinting at a new functional hypothesis for FE which requires further validation through more experiments and analyses.

## Data Availability Statement

The datasets generated for this study can be found in the NCBI-GEO data repository with the accession number: GSE148889 and the link: https://www.ncbi.nlm.nih.gov/geo/query/acc.cgi?acc=GSE148889.

## Ethics Statement

The feed efficiency experiment were approved and carried out in accordance with the Ministry of Environment and Food of Denmark, Animal Experiments Inspectorate under the license number (tilladelsesnummer) 2016-15-0201-01123, and C-permit granted to the principal investigator/senior author (HK).

## Author Contributions

HK conceived and designed the project and obtained funding as the main applicant. VC and HK designed the muscle sampling experiments, phenotype data collection, statistical and bioinformatics analyses. VC performed the sampling, data processing, data visualization, and bioinformatics and statistical analysis. Both authors collaborated in the interpretation of results, discussion, and writing up of the manuscript. Both authors have read, reviewed, and approved the final manuscript.

## Conflict of Interest

The authors declare that the research was conducted in the absence of any commercial or financial relationships that could be construed as a potential conflict of interest.

## Footnotes

1 www.bgi.com
