# Supplementary material for: Genome Regulation and Gene Interaction Networks Inferred From Muscle Transcriptome Underlying Feed Efficiency in Pigs
Source: Front Genet. 2020 Jun 23;11:650. doi: 10.3389/fgene.2020.00650 (PMC7324801; doi:10.3389/fgene.2020.00650)
Supplement: Supplementary file 1 [file Image_1.pdf]

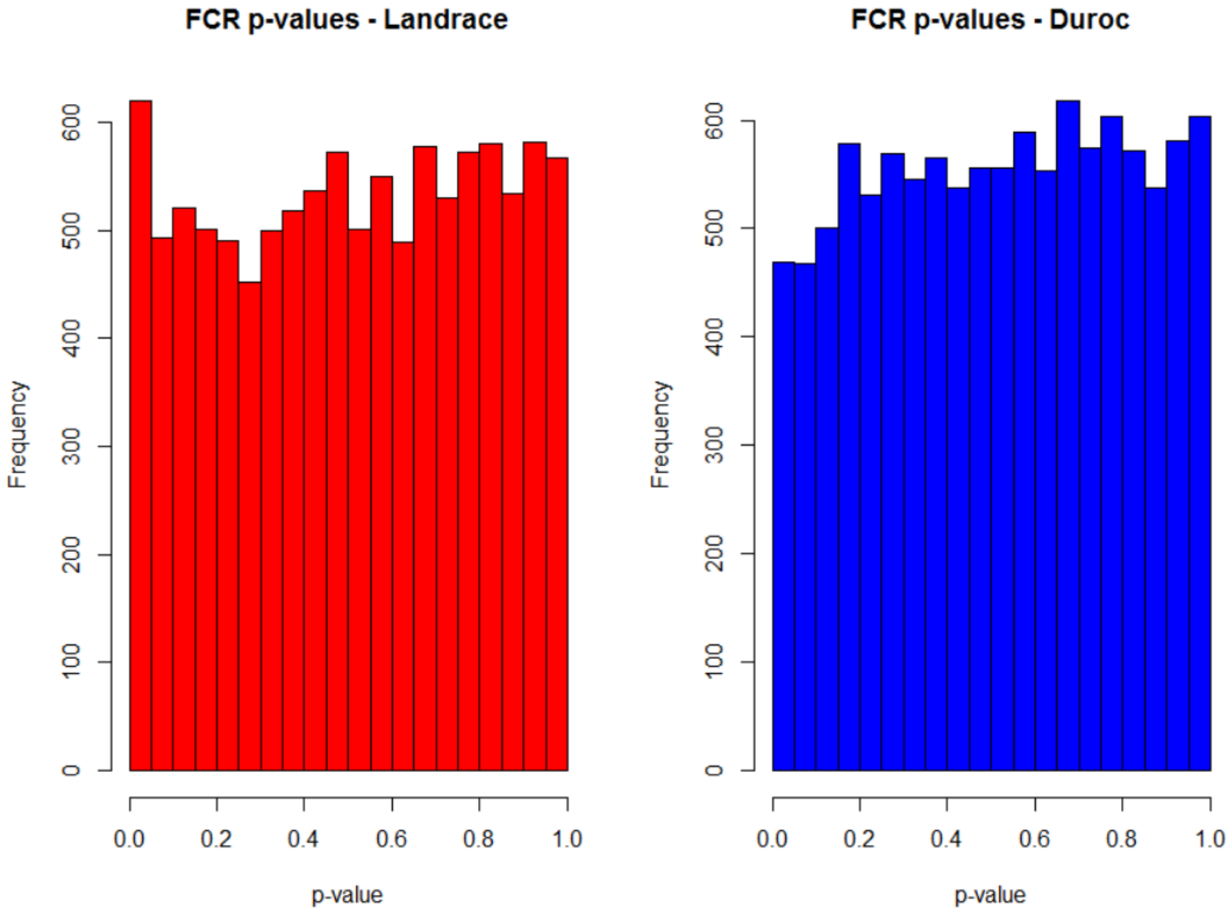

Supplementary figure 1 – Distribution of P-values in the Differential expression analysis for FCR in Danbred Landrace(left) and Danbred Duroc(right)
