# Supplementary material for: Genome Regulation and Gene Interaction Networks Inferred From Muscle Transcriptome Underlying Feed Efficiency in Pigs
Source: Front Genet. 2020 Jun 23;11:650. doi: 10.3389/fgene.2020.00650 (PMC7324801; doi:10.3389/fgene.2020.00650)
Supplement: Supplementary file 2 [file Image_2.PDF]

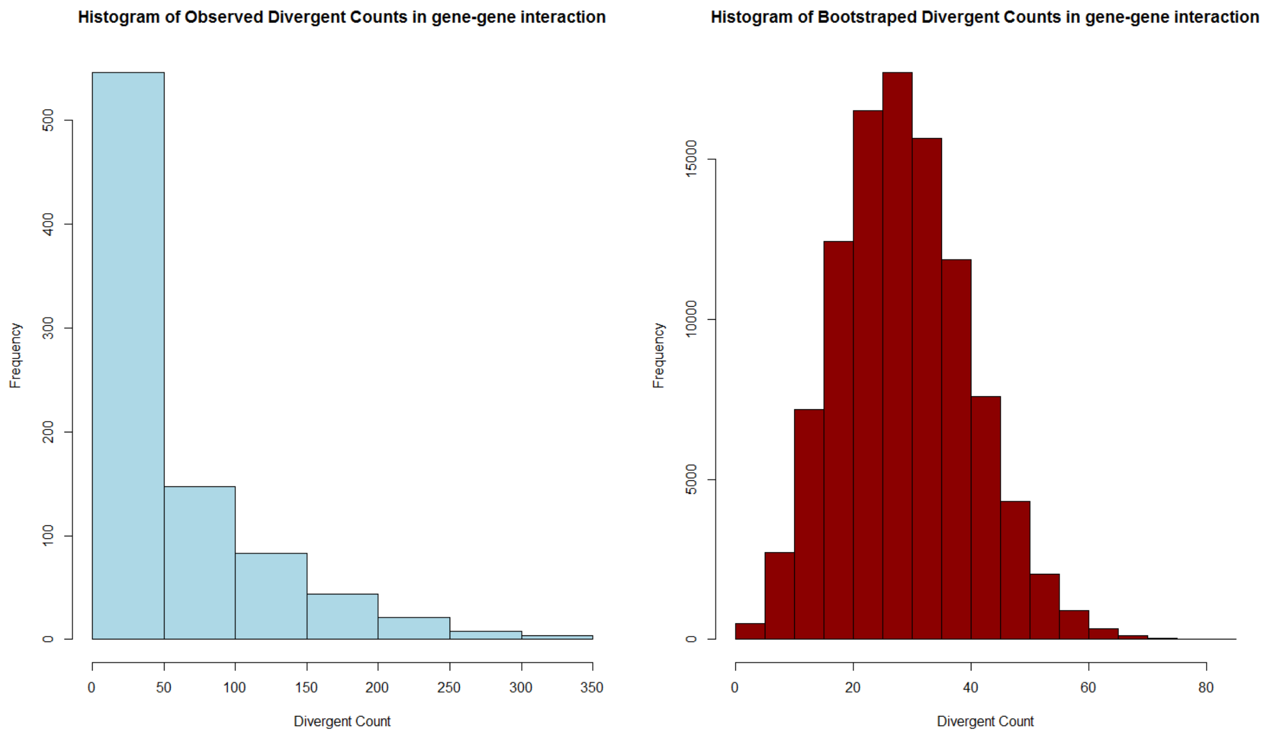

Supplementary figure 2 – Distribution of observed divergent counts for the p-values of the interactions of each of the 853 genes included in the gene-gene interaction anaylsis(left), Distribution of 10^5 divergent counts generated by sampling 853 p-values at a time and calculating the divergent counts from the gene-gene interaction p-values(right). Overall, the empirical and bootstrapped distribution are completely different, indicating we are modelling real interactions.
